# An improved protocol for efficient transformation and regeneration of diverse indica rice cultivars

**DOI:** 10.1186/1746-4811-7-49

**Published:** 2011-12-30

**Authors:** Khirod K Sahoo, Amit K Tripathi, Ashwani Pareek, Sudhir K Sopory, Sneh L Singla-Pareek

**Affiliations:** 1 Plant Molecular Biology, International Centre for Genetic Engineering and Biotechnology, Aruna Asaf Ali Road, New Delhi 110067, India; 2 Stress Physiology and Molecular Biology Laboratory, School of Life Sciences, Jawaharlal Nehru University, New Delhi 110067, India

**Keywords:** rice, transformation, regeneration, mature seed-derived calli, *Agrobacterium*, somatic embryogenesis

## Abstract

**Background:**

Rice genome sequencing projects have generated remarkable amount of information about genes and genome architecture having tremendous potential to be utilized in both basic and applied research. Success in transgenics is paving the way for preparing a road map of functional genomics which is expected to correlate action of a gene to a trait in cellular and organismal context. However, the lack of a simple and efficient method for transformation and regeneration is a major constraint for such studies in this important cereal crop.

**Results:**

In the present study, we have developed an easy, rapid and highly efficient transformation and regeneration protocol using mature seeds as explants and found its successful applicability to a choice of elite indica rice genotypes. We have optimized various steps of transformation and standardized different components of the regeneration medium including growth hormones and the gelling agent. The modified regeneration medium triggers production of large number of shoots from smaller number of calli and promotes their faster growth, hence significantly advantageous over the existing protocols where the regeneration step requires maximum time. Using this protocol, significantly higher transformation efficiency (up to 46%) and regeneration frequency (up to 92% for the untransformed calli and 59% for the transformed calli) were achieved for the four tested cultivars. We have used this protocol to produce hundreds of independent transgenic lines of different indica rice genotypes. Upon maturity, these transgenic lines were fertile thereby indicating that faster regeneration during tissue culture did not affect their reproductive potential.

**Conclusions:**

This speedy, yet less labor-intensive, protocol overcomes major limitations associated with genetic manipulation in rice. Moreover, our protocol uses mature seeds as the explant, which can easily be obtained in quantity throughout the year and kept viable for a long time. Such an easy, efficient and generalized protocol has the potential to be a major tool for crop improvement and gene-function studies on the model monocot plant rice.

## Background

Rice transformation using *Agrobacterium tumefaciens *is a method of choice due to stable and low copy number integration of transfer-DNA (T-DNA) into the plant chromosome and transfer of larger DNA segments with defined ends [1]. Genetic transformation of rice with *Agrobacterium *requires regeneration of an intact plant from a transformed callus and ironically, shoot regeneration represents a major bottleneck in this endeavor. Most of the indica rice genotypes, the world's most cultivated rice types, still remain less amenable to genetic modifications due to their poor regeneration potential. The existing protocols for transformation and regeneration of indica rice are tedious, lengthy, and highly genotype-specific with low efficiency of transformation [1-8]. Considering the significance of genetic transformation in functional genomics and crop improvement, the need of the hour is to develop an easy, rapid, reproducible, widely applicable and highly efficient transformation and regeneration protocol for various indica rice genotypes which does not necessitate further genotype specific standardization.

To achieve the above objective, one needs to consider the factors affecting successful transformation and regeneration thereof. For this, researchers have used either different explants [1,9,10], or changed ratios of different components of the culture medium [2,3], used different gelling agents [8,11], different *Agrobacterium *strains [8], or have even imposed desiccation stress to the calli [8,11,12], yet these protocols suffer from one or the other constraints such as low transformation efficiency, non-availability of rare explants (e.g. immature embryos) throughout the year or genotype specificity etc [1-12].

In the present study, we have developed a highly efficient and reproducible *A. tumefaciens *mediated transformation protocol using mature seeds as explants. To significantly improve the regeneration frequency, we optimized the kind and concentration of the gelling agent, proportion of growth regulators and period of dark incubation and studied their effect individually. We then came up with a comprehensive protocol where all these modifications were combined to attain maximum transformation efficiency. We show generalized application of our protocol on four diverse, until now, recalcitrant indica rice cultivars. Very high regeneration frequency of transformed as well as untransformed calli was obtained here, thus overcoming the main hurdle in genetic manipulation of rice.

## Results and Discussion

### Overview of the modified protocol for efficient transformation and regeneration

**Figure 1:**
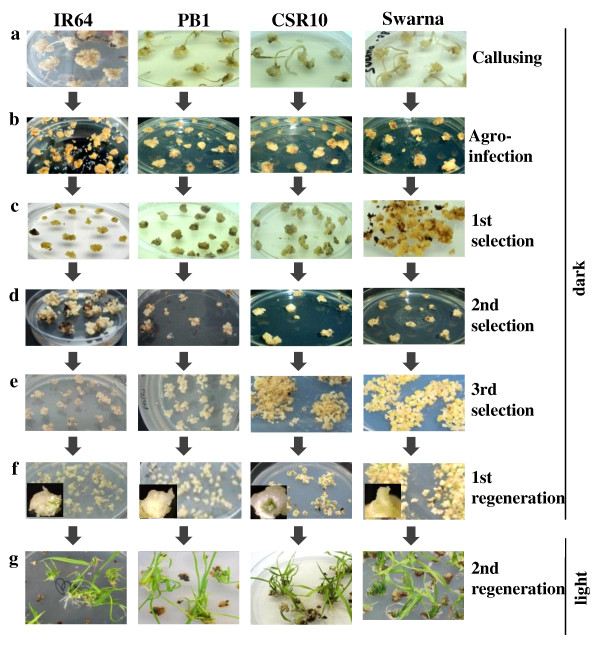
**Steps for *Agrobacterium *mediated transformation and regeneration of rice calli**. Four indica rice cultivars i.e. IR64, PB1, CSR10 and Swarna were used here. Steps a-f have to be performed under dark conditions. (**a**) Callus initiation on callus induction medium. (**b**) Agro-infection and co-cultivation of calli with *Agrobacterium tumefaciens*. (**c, d & e**) First, second and third selection cycles of transformed calli/microcalli in presence of hygromycin (50 mg/l). (**f**) Morphology of microcalli after first phase of regeneration. Inset shows somatic embryo for each of the rice cultivar. (**g**) Regeneration of large number of shoots from microcalli during second phase of regeneration under light condition.

**Figure 2:**
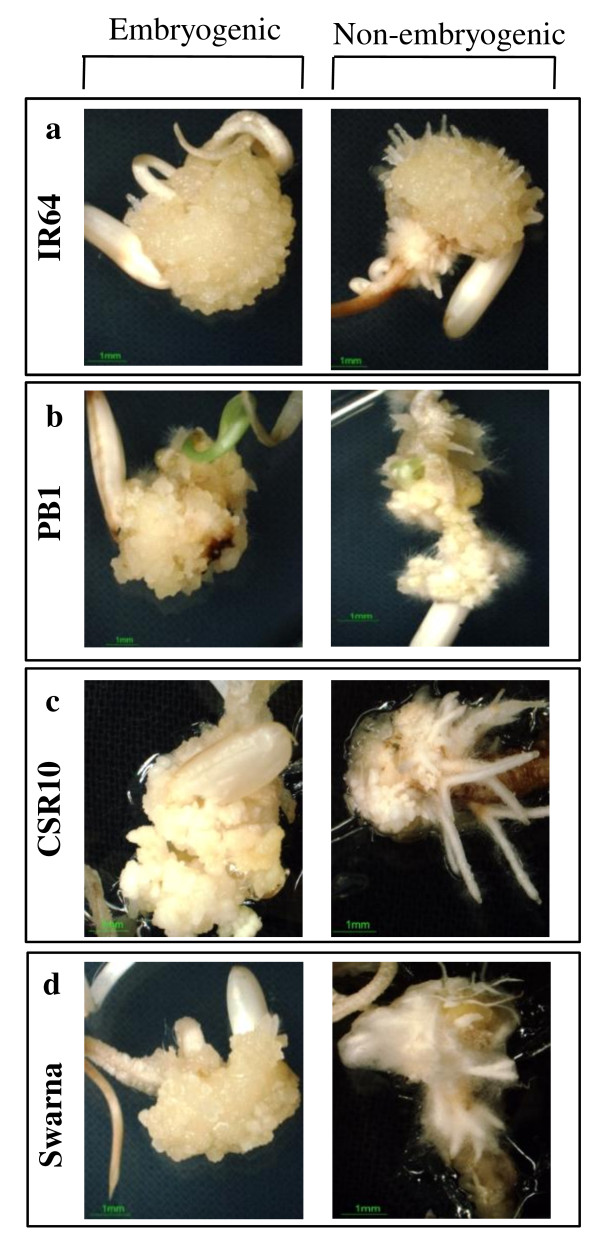
**Morphological distinction between embryogenic and non-embryogenic calli of rice**. Left panel shows embryogenic whereas the right panel shows non-embryogenic calli of different indica rice cultivars viz. IR64, PB1, CSR10 and Swarna.

**Figure 3:**
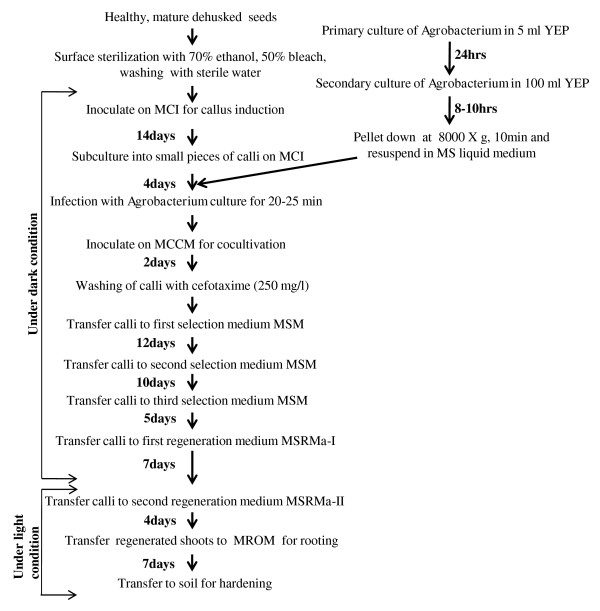
**Schematic workflow of the protocol**. The flow chart shows the essential steps followed in the protocol. See Methods section for details including composition of different media.

We selected an elite rice cultivar i.e. IR64 for initial standardization of various factors critical for its enhanced transformation and regeneration. The optimized protocol was then extended to other indica cultivars such as CSR10, Pusa Basmati 1 (PB1) and Swarna to develop a transformation and regeneration method that is widely applicable. The basis of selection for these genotypes is either their wide cultivation and high yield capacity (IR64 and Swarna) or stress tolerance (CSR10) or high economic value (PB1). We have used seeds as the explants, as these would be available to the researchers all round the year. Almost 99-100% seeds developed scutellar calli (Figure 1a) within 14 days of inoculation on our modified callus induction medium-MCI (see Methods section). However, for further sub-culturing, only the embryogenic calli were subcultured for 4 days on fresh MCI medium (Figure 2). The embryogenic callusing efficiency in all the four genotypes was found to be between 92-97% (Additional File 1 and 2). We observed that scrutiny of embryogenic calli from non-embryogenic ones at this step was essential as this would affect the transformation and regeneration capability of the calli. After 4 days, these subcultured calli were subjected to Agro-infection using *Agrobacterium *carrying the gene construct (shown in Additional File 3) and co-cultivated for ~48 hours on co-cultivation medium-MCCM (see Methods section). Once the growth of *Agrobacterium *could be visualized at the periphery of the individual calli (Figure 1b), these were shifted to selection medium-MSM (see Methods section). After ~12 days on the MSM medium, some of the calli turned brownish while the other remained creamish (Figure 1c). The creamish colored calli were then transferred to fresh MSM medium for a second selection cycle where small microcalli started growing on the mother calli (Figure 1d). These microcalli were gently separated from the mother calli and transferred to fresh MSM medium for the third selection (Figure 1e). This step allowed the proliferation of microcalli which were then shifted to regeneration medium I (MSRM-I) and maintained in dark for 7 days (see Methods section). Here, most of the microcalli developed into somatic embryos (Figure 1f and inset). These were shifted to second regeneration medium-MSRM-II (see Methods section) and placed in light for 4 days where "green-spots" appeared which subsequently developed into shoots (Figure 1g). Shifting of microcalli from dark to light was found to be crucial as microcalli maintained under continuous dark conditions for regeneration resulted in the development of much elongated albino plantlets. These albino plantlets developed only into thinner and weaker shoots after exposure to light. Another experiment was carried out where the microcalli were shifted to regeneration medium and incubated in light (16 h light/8 h dark) from the beginning itself. Under these conditions, we observed that the number of days required for regeneration was significantly more and also the frequency of regeneration was very low (data not shown). The essential steps described above have been summarized in the form of a flow chart (Figure 3).

### Optimization of different components of the regeneration medium

During our experimentation, we found that different growth hormones and the type of gelling agent as well as their concentration used in the regeneration medium are important factors influencing the regeneration frequency. To obtain a higher regeneration frequency for all the four indica rice cultivars, we standardized for the optimum concentration and composition of growth hormones. One type of regeneration medium (MSRMa) contained two hormones viz. kinetin and naphthalene acetic acid whereas the other (MSRMb) contained three hormones viz. 6-benzylaminopurine, kinetin and naphthalene acetic acid (see Methods section). We also standardized for the gelling agent and its optimum concentration in the regeneration medium. Different gelling agents such as phytagel, agar, mixture of phytagel and agar, and agarose were used. The regeneration frequency for the tested cultivars, ranged between 20-30% when either phytagel or agar was used (Additional File 4). However, when the combination of phytagel and agar was used, a slightly higher regeneration frequency (23-36%) was obtained. Importantly, significantly higher regeneration frequency (54-77%) was obtained when 0.8% agarose was used as the gelling agent (Additional File 4). These observations were found to be statistically significant as revealed by one-way analysis of variance (ANOVA, *P *< 0.05).

**Figure 4:**
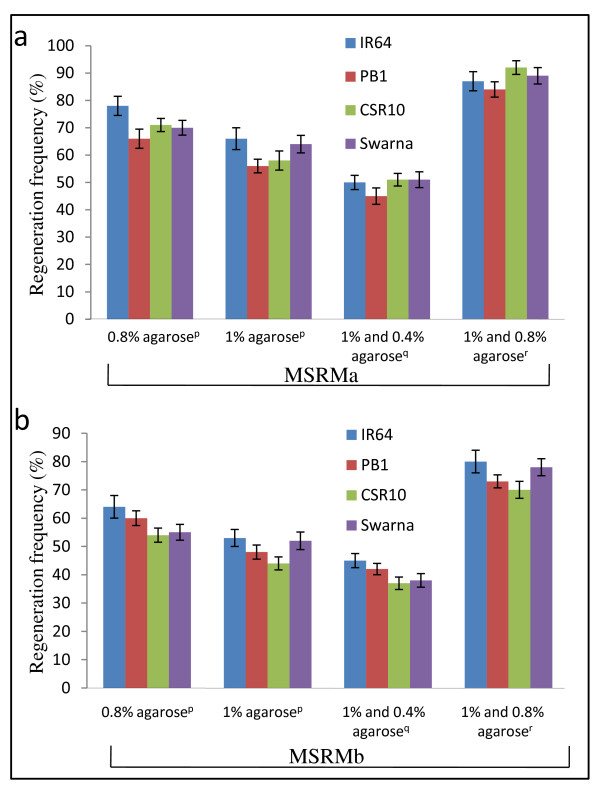
**Effect of growth regulators and gelling agents during regeneration**. Regeneration frequency (%)* of four rice cultivars; IR64, PB1, CSR10 and Swarna using MSRMa (**a**) or MSRMb (**b**) medium containing varying concentrations of agarose. Data shown are mean ± standard deviation of three independent experiments. For each cultivar, the statistical significance of the effects of different concentrations of agarose and the media components (MSRMa or MSRMb) on regeneration frequency was tested using two-way ANOVA (*P *< 0.0001) followed by Tukey HSD test (HSD_0.5_). *Regeneration Frequency (%) = no. of microcalli regenerating shoots/no. of microcalli incubated × 100%. ^p^Agarose concentration used throughout the regeneration i.e. in both the phases of regeneration. ^q^1% agarose was used during the first phase of regeneration and 0.4% in the second phase. ^r^1% agarose was used during the first phase of regeneration and 0.8% in the second phase.

Once it was found that agarose is the most appropriate gelling agent during regeneration, we optimized its concentration for regeneration using both MSRMa and MSRMb medium. Here, the experiment was conducted in four identical sets for each of the MSRMa or MSRMb medium (Additional File 5). In the first set, 0.8% agarose was used as the gelling agent to prepare the medium and the callus was transferred and maintained on this media in dark for a week followed by shifting the calli to light till the appearance of the shoots. In the second set, 1% agarose was used in place of 0.8% and the experiment was performed similar to the first set. In the third set, the regeneration medium was initially prepared with 1% agarose and the calli were transferred to this media and maintained in dark for 1 week. These calli were subsequently transferred to light on the same regeneration media but gellified with 0.4% agarose. In the fourth set, the initial concentration of agarose in the regeneration medium was kept as 1% for 1 week in dark and was reduced to 0.8% before shifting to light. The regeneration frequency obtained on MSRMa (Figure 4a) or MSRMb (Figure 4b) with different concentrations of agarose have been shown along with the experimental details in Additional File 5. All the four genotypes responded best to the fourth set of MSRMa (regeneration medium containing kinetin and naphthalene acetic acid gellified with 1% agarose and subsequently with 0.8% agarose). The shoot regeneration frequency on this medium ranged from 84-92% (Figure 4a) while the corresponding values on MSRMb were 52-79% (Figure 4b). The statistical significance of the above dataset was analyzed using two-way ANOVA (*P *< 0.0001) followed by Tukey HSD test which showed that regeneration frequency was highly affected by the media composition as well as the percentage of agarose used in the culture media. Henceforth, MSRMa gellified with 1% agarose (MSRMa-I) and subsequently with 0.8% agarose (MSRMa-II) was used in further studies.

A higher concentration of agarose imposes desiccation stress to the microcalli [11]. In the present study, it was observed that in the first phase of regeneration, a higher concentration of agarose (1%) promotes the formation of somatic embryos [13] under dark conditions within a period of 6-7 days (Figure 1f, inset). Regeneration in dark is a critical step because somatic embryogenesis is promoted by auxins which are degraded more quickly under light conditions [14]. During the second phase of regeneration, slight reduction in the concentration of agarose (optimally 0.8%, MSRMa-II) triggered a faster appearance of large number of green spots within 1-2 days time which developed into proper shoots, when the calli transferred to this medium were kept under light (Figure 1g). Using a higher or lower concentration of the gelling agent-agarose in this phase also, could promote shoot regeneration but the frequency was lower (Figure 4a). This modification in the medium helped in obtaining large number of regenerated plantlets from smaller number of calli in a short period of time, thereby resulting in cutting down the total duration of the protocol significantly.

**Figure 5:**
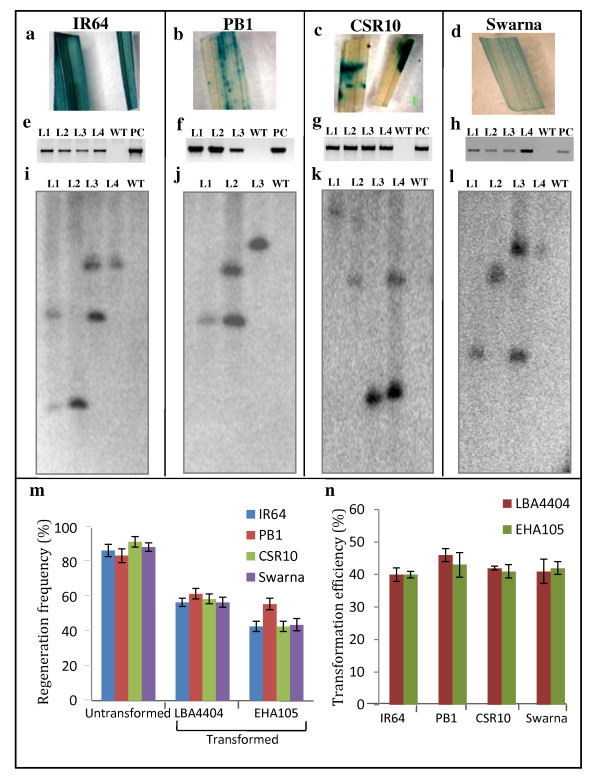
**Confirmation of transgenic status of the regenerated rice plants**. GUS expression in leaves (**a-d**); PCR amplification of transgene from genomic DNA (**e**-**h**) and Southern analysis (**i-l**) of rice cultivars IR64, PB1, CSR10 and Swarna respectively. For Southern analysis, genomic DNA was restricted with a single cutter enzyme (*Xba*I) of T-DNA to indicate the copy number of the transgene and independence of the events. (**m**) Regeneration frequency (%)^p ^of untransformed calli (regenerated without co-cultivation with *Agrobacterium*) and transformed calli using either LBA4404 or EHA105. (**n**) Transformation efficiency (%)^q ^of IR64, PB1, CSR10 and Swarna using different *Agrobacterium *strains viz. LBA4404 or EHA105. Error bars, standard deviations; n = 3. ^p^Regeneration Frequency (%) = no. of microcalli regenerating shoots/no. of microcalli incubated × 100%. ^q^Transformation efficiency (%) = no. of plants expressing GUS/no. of calli inoculated with *Agrobacterium *X 100%

We found the regeneration frequency to range between 84-92% for the untransformed calli and 42-59% for the transformed calli for all the cultivars (Figure 5m). Therefore, the kind of gelling agent and its concentration, the proportion of growth regulators, time duration and regeneration in dark followed by that in light, all collectively influenced the regeneration frequency. Higher regeneration frequency, of the transformed calli, for different indica rice genotypes would resolve the major hurdle in efficient genetic transformation of rice via tissue culture.

### Molecular confirmation of the transgenic status of regenerated plants

After obtaining large number of regenerated shoots, these were subsequently shifted to the rooting medium-MROM (see Methods section) which led to development of normal roots. At maturity, the regenerated plants (T_0_) exhibited normal morphology and growth and were fertile. These plants were then analyzed for their transgenic status by screening for the expression of reporter-GUS (Figure 5a-d). The plants showing the expression of GUS were further confirmed for transgene integration by PCR analysis (Figure 5e-h). Southern analysis of these plants with single cutter enzyme (*Xba*I) between left and the right border of T-DNA confirmed them to be either single- or two- copy integrations. This analysis also confirmed these transgenic events to be completely independent of each other (Figure 5i-l). Furthermore, we found that usage of different *A. tumefaciens *strains (LBA4404 or EHA105) did not affect the transformation efficiency to a large extent and it was found to range between 40-46% in the four rice genotypes (Figure 5n, Additional File 1 and 2).

Taken together, the protocol presented here has the following significant advantages over the methods that are currently available; higher regeneration frequency (84-92%) and transformation efficiency (40-46%) especially in hardy indica rice cultivars such as IR64 and CSR10 etc., wide applicability, one can obtain large number of shoots from smaller number of calli and shorter duration of the protocol in totality. In literature, so far the highest transformation frequency (number of independent hygromycin-resistant and GUS-positive plants per embryo) has been reported to be around 30% using immature rice embryos as the explants [10]. However, usage of such explants poses several difficulties due to their tedious preparation procedures. In a later study, it has been discussed that the quality of rice embryo is a critical determinant of successful transformation and obtaining 'good' embryos requires healthy plants at the right developmental stage growing in a properly-conditioned environment with respect to temperature, day-length and light intensity [1]. Maintenance of all these conditions in laboratory is laborious and highly expensive. However, the protocol presented here uses mature seeds as the starting material i.e. explant and does away with such limitations because mature seeds can easily be obtained in quantity throughout the year and kept viable for a long time. Moreover, our protocol yields a higher transformation efficiency of ~45% despite using mature seed-derived calli as the target material for transformation. A protocol with higher transformation efficiency would facilitate functional genomics of rice and help in achieving biotechnological goals.

### Trouble-shooting

Although the method is quite easy and reproducible, there are some aspects that should be considered while trouble-shooting.

#### No callus induction

• Change the seed lot if it is an old one and take only intact healthy seeds.

• Surface sterilization (with 70% ethanol) of seed should not be done for more than 90 seconds.

• Use freshly prepared plant hormones during preparation of callus induction medium.

• Maintain the inoculated seeds in the petriplates in dark during callus induction period.

#### Growth of *Agrobacterium *on the calli during selection

• Do not grow the *Agrobacterium *beyond an O.D_600 _of 1.0 in YEP medium.

• Incubation of calli during co-cultivation should not be more than 48 hours in co-cultivation medium-MCCM.

• Wash the calli thoroughly with sterile water containing 250 mg/l cefotaxime after 2 days of co-cultivation.

• Use freshly prepared cefotaxime for selection medium preparation and during washing.

• Add cefotaxime and hygromycin in the culture medium after autoclaving once its temperature reaches around 48°C.

#### No proliferation of calli in MSM

• Confirm the selection marker gene sequence in the construct.

• Ensure the right type and concentration of antibiotic in the selection medium.

#### Low regeneration frequency

• Check the concentrations of gelling agent and plant hormones in the regeneration medium MSRMa.

• Use only freshly prepared plant hormones.

• Dry or remove moisture and water droplets in the culture petriplate containing regeneration medium before inoculation.

• Proper temperature (28 ± 2^°^C) should be adjusted during the culture conditions.

#### Low transformation efficiency

• Use only embryogenic scutellar calli for Agro-infection.

• Always use freshly prepared acetosyringone.

• Incubation of the embryogenic scutellar calli should not be more or less than 20-30 minutes during Agro-infection.

• Avoid shifting brown or black calli during subsequent transfer to selection medium.

## Conclusions

Regeneration of the transformed calli is considered to be a major obstacle in genetic transformation of rice. Several laboratories around the globe are making a serious effort towards breaking this obstacle. With our modified protocol, we have made a significant advance in enhancing both the transformation efficiency as well as regeneration frequency of various indica rice genotypes which will be of great help to rice studies. Our optimization of the kind and concentration of the gelling agent, proportion of growth regulators and period of dark incubation led to the formulation of a comprehensive and improved protocol. This protocol has desirable advantages such as high transformation efficiency (~45%), high regeneration frequency (~90%) and shorter duration of total protocol (~2 months) than any other protocol reported for indica rice so far [1,15]. Moreover, usage of mature seeds as the explants renders an extra advantage due to their unlimited availability throughout the year. The protocol works with almost same efficiency with all the tested indica genotypes doing away with the genotype-specific optimizations. Thus, it has the potential to serve as a simple, easy and efficient protocol for rice transformation paving the way for gene function studies on the model monocot plant-rice as well as for easier production of improved genotypes by genetic engineering.

## Methods

### Plant material and embryogenic callus induction

Mature dry seeds of four indica rice cultivars; IR64, CSR10, PB1 and Swarna were used in this study. Scutellar embryogenic calli were used as the target material for optimization of transformation and regeneration. For this purpose, healthy seeds of all the four cultivars were surface-sterilized with 70% ethanol (v/v) for 1 min, followed by 30 min in 50% (v/v) commercial bleach with shaking at 180 rpm. Seeds were then washed 8-10 times with sterile distilled water and dried on autoclaved Whatman paper (3 mm) for 5 min. For callus induction, twelve to thirteen seeds were inoculated per petriplate on callus induction medium (MCI) and incubated at 27 ± 1°C in dark. MCI was prepared using basal MS salts containing all vitamins (Caisson laboratories, Catalog no. MSP09) [16] supplemented with 30 g/l maltose, 0.3 g/l casein hydrolysate, 0.6 g/l L-proline, 3.0 mg/l 2,4-dichlorophenoxyacetic acid (2,4-D), 0.25 mg/l 6-benzylaminopurine (BAP), gelled with 3.0 g/l phytagel and pH adjusted to 5.8 before autoclaving. After 14 days in dark, non-embryogenic calli (compact, non-friable calli that develop root like structures) were discarded and only embryogenic calli (Figure 2) were selected. These embryogenic calli were cut into approximately 3 equal halves and subcultured again onto fresh MCI and kept for 4 days (dark, 27 ± 1°C) before transformation with *Agrobacterium tumefaciens*.

### *Agrobacterium *strains and construct used for transformation

The transgene used in this study is the glyoxalase I (Bj*glyI*) gene from *Brassica juncea *(GenBank accession no. Y13239). This was cloned in pCAMBIA1304 plant transformation vector at *Xba*I and *Kpn*I restriction sites and the gene construct (Additional File 3) was finally transformed in two commonly used strains of *Agrobacterium tumefaciens*; LBA4404 and EHA105. This vector has *nptII *(neomycin phosphotransferase) and *hptII *(hygromycin phosphotransferase) genes as the selectable markers for bacteria and plants respectively. It has both *uidA *(for GUS) and green fluorescent protein (mGFP) as the reporter genes.

### Preparation of *Agrobacterium *culture

Primary culture of *Agrobacterium *was prepared by inoculating single colony from a freshly streaked plate, in 5 ml of autoclaved liquid YEP medium (10 g/l bactopeptone, 10 g/l yeast extract, 5 g/l sodium chloride, pH 7.0) supplemented with 25 mg/l streptomycin, 10 mg/l rifampicin, 50 mg/l kanamycin. The culture was incubated for 16-20 h on a rotatory incubator shaker (Kuhner, Switzerland) at 200 rpm in dark at 28°C. Secondary culture was prepared in a 500 ml baffled flask containing 100 ml YEP medium (supplemented with same antibiotics as used for primary culture) by adding 0.4% of the primary culture and grown under similar conditions. Once the O.D. _600 _reached ~1.0, *Agrobacterium *cells were pelleted by centrifugation at 8000 × g for 15 min at 4°C. The cells were resuspended in MS resuspension medium containing 150 μM acetosyringone (MS salts, 68 g/l sucrose, 36 g/l glucose, 3 g/l KCl, 4 g/l MgCl_2_, pH 5.2) to adjust the O.D._600 _of the bacterial suspension to 0.3.

### Co-cultivation and selection of transformed calli

The 4 day subcultured embryogenic calli were collected and Agro-infected by immersing them in the *Agrobacterium *culture (LBA4404 or EHA105) for 20-25 min with intermittent gentle shaking at 50 rpm. The Agro-infected calli were dried on sterile Whatman No. 3 filter paper for 5 min. Calli were then transferred to the co-cultivation medium (MCCM)-MCI containing 10 g/l glucose, pH 5.2, 150 μM acetosyringone [17] and incubated at 27 ± 1°C in the dark for around 48 hours. Once slight growth of *Agrobacterium *appeared around most of the calli, the calli were rinsed 8-10 times with 250 mg/l cefotaxime in sterile distilled water, dried on sterile Whatman No. 3 filter paper and transferred onto first selection medium-MSM (MCI containing 250 mg/l cefotaxime and 50 mg/l hygromycin) and incubated for 12 days at 27 ± 1°C in dark. After the first selection, brown or black calli were removed and only creamish healthy calli were shifted to the fresh MSM media for second selection and maintained at 27 ± 1°C in dark. After second selection for 10 days, microcalli could be observed which were finally transferred to fresh MSM media for third selection and allowed to proliferate for 5 days at 27 ± 1°C in dark.

### Regeneration of transformed calli

After third selection, black or brown microcalli were discarded and only granular 'macrocalli' were transferred onto two different media containing either two or three growth regulators viz. MSRMa and MSRMb. MSRMa comprised of MS salts, 30 g/l maltose, 2 mg/l kinetin, 0.2 mg/l naphthalene acetic acid (NAA), pH 5.8; 250 mg/l cefotaxime and 30 mg/l hygromycin added after autoclaving. While MSRMb consisted of MS salts, 30 g/l maltose, 2.7 mg/l BAP, 1.2 mg/l kinetin, 0.5 mg/l NAA, pH 5.8; 250 mg/l cefotaxime and 30 mg/l hygromycin added after autoclaving. Both MSRMa and MSRMb were supplemented with either 8 or 10 g/l agarose during the first phase and 4, 8 or 10 g/l agarose in the second phase of regeneration. These microcalli were incubated at 27 ± 1°C in dark for 7 days for the first phase of regeneration. During the second phase of regeneration, these were shifted to fresh regeneration medium with different concentrations of agarose (as described above and in Results section) and incubated in light for 4 days. The regeneration frequency was calculated as per the formula given below [18].

$$\text{Regeneration frequency}\left(\%\right)=\frac{\text{Number of microcalli regenerating shoots}}{\text{Number of microcalli incubated}}\times 100\%$$

For development of roots, the regenerated shoots were shifted to jam bottles containing rooting medium MROM (comprising half strength MS salts, 30 g/l sucrose, 3.0 g/l phytagel, pH 5.8; 250 mg/l cefotaxime and 30 mg/l hygromycin added after autoclaving) and maintained at 27 ± 1°C in light for a week.

### Statistical analysis

Statistical analyses were performed by either one-way or two-way ANOVA, as the case may be, individually for each cultivar and the *P *value was obtained. Post-ANOVA comparisons were carried out by Tukey HSD test (http://faculty.vassar.edu/lowry/anova2u.html) and the differences between means were compared with HSD_0.5 _values obtained for the particular dataset.

### Molecular confirmation of putative transgenic plants

Histochemical staining of GUS expression in leaf samples was performed as described previously [19]. The transformation efficiency was calculated as per the formula given below [18].

$$\text{Transformation efficiency }\left(\%\right)=\frac{\text{Number of GUS positive plants}}{\text{Number of calli inoculated with }Agrobacterium}\times 100\%$$

To confirm the presence of transgene in GUS positive plants, we analyzed these plants along with wild type (WT) lines through PCR analysis. Total genomic DNA from various independent transgenic lines was extracted as described elsewhere [20] and was used for PCR using the Bj*GlyI *specific primers (forward- 5'ATGGCGTCGGAAGCGAAGG3' and reverse- 5'TCAAGCTGCGTTTCCGGCTG3'). The PCR products were analyzed on 1% agarose gel containing ethidium bromide and visualized under gel documentation unit. The PCR-positive plants were analyzed by Southern blot analysis for the integration of *GlyI *gene in the rice genome. For this, 20 μg of genomic DNA isolated from WT as well as GUS and PCR positive plants, was restricted with a single cutter of T-DNA - *Xba*I. The digestion products were resolved on a 0.8% agarose gel and subsequently blotted onto nylon membrane and cross-linked with UV light. The membrane was probed with α^32^P-dCTP radiolabeled Bj*GlyI *gene using standard protocol and visualized in phosphorimager.

### Composition of medium used at various stages

#### MCI medium

MS basal salts and vitamins (Caisson laboratories, Catalog no. MSP09) supplemented with 30 g/l maltose, 0.3 g/l casein hydrolysate, 0.6 g/l L-proline, 3.0 mg/l 2, 4-D, 0.25 mg/l BAP, pH 5.8 and 3 g/l phytagel.

#### YEP medium

10 g/l bactopeptone, 10 g/l yeast extract, 5 g/l sodium chloride, pH 7.0.

#### MS resuspension medium

MS salts (MSP09), 68 g/l sucrose, 36 g/l glucose, 3 g/l KCl, 4 g/l MgCl_2_, pH 5.2 and 150 μM acetosyringone (freshly prepared at a concentration of 1 M in 100% Dimethyl sulfoxide).

#### MCCM

MS salts (MSP09) supplemented with 30 g/l maltose, 0.3 g/l casein hydrolysate, 0.6 g/l L-proline, 10 g/l glucose, 3 mg/l 2, 4-D, 0.25 mg/l BAP, pH 5.2, 3 g/l phytagel and 150 μM acetosyringone added after autoclaving.

#### MSM

MS salts (MSP09) supplemented with 30 g/l maltose, 0.3 g/l casein hydrolysate, 0.6 g/l L-proline, 3 mg/l 2,4-D, 0.25 mg/l BAP, pH 5.8, 3 g/l phytagel; 250 mg/l cefotaxime and 50 mg/l hygromycin added after autoclaving.

#### MSRMa-I

MS salts (MSP09) supplemented with 30 g/l maltose, 2 mg/l kinetin, 0.2 mg/l NAA, pH 5.8, 10 g/l agarose; 250 mg/l cefotaxime and 30 mg/l hygromycin was added after autoclaving.

#### MSRMa-II

MS salts (MSP09) supplemented with 30 g/l maltose, 2 mg/l kinetin, 0.2 mg/l NAA, pH 5.8, 8 g/l agarose; 250 mg/l cefotaxime and 30 mg/l hygromycin added after autoclaving.

#### MSRMb

MS salts (MSP09) supplemented with 30 g/l maltose, 2.7 mg/l BAP, 1.2 mg/l kinetin, 0.5 mg/l NAA, pH 5.8, 4 or 8 or 10 g/l agarose; 250 mg/l cefotaxime and 30 mg/l hygromycin added after autoclaving.

#### MROM

Half strength MS salts (MSP09), 30 g/l sucrose, pH 5.8, 3 g/l phytagel; 250 mg/l cefotaxime and 30 mg/l hygromycin added after autoclaving.

## Competing interests

The authors declare that they have no competing interests.

## Authors' contributions

KKS and SLS-P performed the experiments. AKT participated in the experiments and writing of the manuscript. SKS, SLS-P and AP conceptualized the study and participated in the preparation of the manuscript. All authors have read and approved the final manuscript.

## Supplementary Material

Additional file 1 **Transformation efficiency (LBA4404 mediated) of various indica rice cultivars**. Table showing transformation efficiency (LBA4404 mediated) of different indica rice cultivars viz. IR64, PB1, CSR10 and Swarna. R1, R2 and R3 represent three replicates of the experiment. Click here for file

Additional File 2 **Transformation efficiency (EHA105 mediated) of various indica rice cultivars**. Table showing transformation efficiency (EHA105 mediated) of different indica rice cultivars viz. IR64, PB1, CSR10 and Swarna. R1, R2 and R3 represent three replicates of the experiment. Click here for file

Additional File 3 **Gene construct used for rice transformation**. Schematic representation of the gene construct which shows Bj*GlyI *cloned in pCAMBIA1304 plant transformation vector and used for *Agrobacterium *mediated rice transformation. Click here for file

Additional File 4 **Optimization of the kind of gelling agent during regeneration**. Table showing regeneration frequency using different gelling agents viz. agar, phytagel, phytagel and agar together, and agarose during regeneration in MSRMa and MSRMb. Click here for file

Additional File 5 **Optimization of proportion of hormones and agarose concentration during different phases of regeneration**. Table showing regeneration frequency using various agarose concentrations in MSRMa (A) and MSRMb (B). Click here for file
