# Supplementary material for: An improved protocol for efficient transformation and regeneration of diverse indica rice cultivars
Source: Plant Methods. 2011 Dec 30;7:49. doi: 10.1186/1746-4811-7-49 (PMC3284416; doi:10.1186/1746-4811-7-49)
Supplement: Additional file 1 — Transformation efficiency (LBA4404 mediated) of various indica rice cultivars. Table showing transformation efficiency (LBA4404 mediated) of different indica rice cultivars viz. IR64, PB1, CSR10 and Swarna. R1, R2 and R3 represent three replicates of the experiment. [file 1746-4811-7-49-S1.PDF]

Additional file 1: Transformation efficiency (LBA4404 mediated) of various indica rice cultivars. Table showing transformation efficiency (LBA4404 mediated) of different indica rice cultivars viz. IR64, PB1, CSR10 and Swarna. R1, R2 and R3 represent three replicates of the experiment.

Cultivar	Replicates	No. of seeds inoculated	No. of embryogenic calli generated	Total no. of calli used for transformation [a]	No. of plantlets regenerated roots	No. of plants expressing GUS	Transformation Efficiency (%) [b]
IR64	R1	150	140	420	163	159	38
	R2	180	175	525	225	216	41
	R3	200	191	573	242	241	42
PB1	R1	180	173	519	245	237	46
	R2	200	190	570	255	249	44
	R3	170	162	486	260	234	48
CSR10	R1	200	192	576	239	231	40
	R2	190	185	555	243	238	43
	R3	210	201	603	274	268	44
Swarna	R1	200	194	582	233	227	39
	R2	150	141	323	136	130	40
	R3	180	172	516	247	239	46

[a] The embryogenic calli generated after seed-inoculation were sub-cultured and used for transformation.

[b] Transformation efficiency (%) = No. of plants expressing GUS/No. of calli inoculated with Agrobacterium X 100%
