# Supplementary material for: An improved protocol for efficient transformation and regeneration of diverse indica rice cultivars
Source: Plant Methods. 2011 Dec 30;7:49. doi: 10.1186/1746-4811-7-49 (PMC3284416; doi:10.1186/1746-4811-7-49)
Supplement: Additional File 2 — Transformation efficiency (EHA105 mediated) of various indica rice cultivars. Table showing transformation efficiency (EHA105 mediated) of different indica rice cultivars viz. IR64, PB1, CSR10 and Swarna. R1, R2 and R3 represent three replicates of the experiment. [file 1746-4811-7-49-S2.PDF]

**Additional file 2: Transformation efficiency (EHA105 mediated) of various indica rice cultivars.** Table showing transformation efficiency (EHA105 mediated) of different indica rice cultivars viz. IR64, PB1, CSR10 and Swarna. R1, R2 and R3 represent three replicates of the experiment.

| Cultivar | Replicates | No. of seeds inoculated | No. of embryogenic calli generated | Total no. of calli used for transformation [a] | No. of plantlets regenerated roots | No. of plants expressing GUS | Transformation Efficiency (%) [b] |
|---|---|---|---|---|---|---|---|
| IR64 | R1 | 160 | 150 | 450 | 196 | 181 | 40 |
| | R2 | 100 | 92 | 276 | 134 | 108 | 39 |
| | R3 | 200 | 189 | 567 | 242 | 235 | 41 |
| PB1 | R1 | 100 | 91 | 269 | 136 | 126 | 47 |
| | R2 | 150 | 143 | 429 | 184 | 172 | 40 |
| | R3 | 100 | 94 | 282 | 128 | 117 | 41 |
| CSR10 | R1 | 100 | 90 | 270 | 122 | 116 | 43 |
| | R2 | 150 | 139 | 417 | 181 | 163 | 39 |
| | R3 | 170 | 155 | 465 | 203 | 199 | 42 |
| Swarna | R1 | 150 | 142 | 426 | 180 | 176 | 41 |
| | R2 | 150 | 140 | 420 | 169 | 165 | 39 |
| | R3 | 100 | 94 | 282 | 131 | 122 | 43 |

[a] The embryogenic calli generated after seed-inoculation were sub-cultured and used for transformation.

[b]Transformation efficiency (%) = No. of plants expressing GUS/No. of calli inoculated with Agrobacterium X 100%
