# Supplementary material for: An improved protocol for efficient transformation and regeneration of diverse indica rice cultivars
Source: Plant Methods. 2011 Dec 30;7:49. doi: 10.1186/1746-4811-7-49 (PMC3284416; doi:10.1186/1746-4811-7-49)

Additional file 3: **Gene construct used for rice transformation.**

Schematic representation of the gene construct which shows Bj*GlyI* cloned in pCAMBIA1304 plant transformation vector and used for Agrobacterium mediated rice transformation.

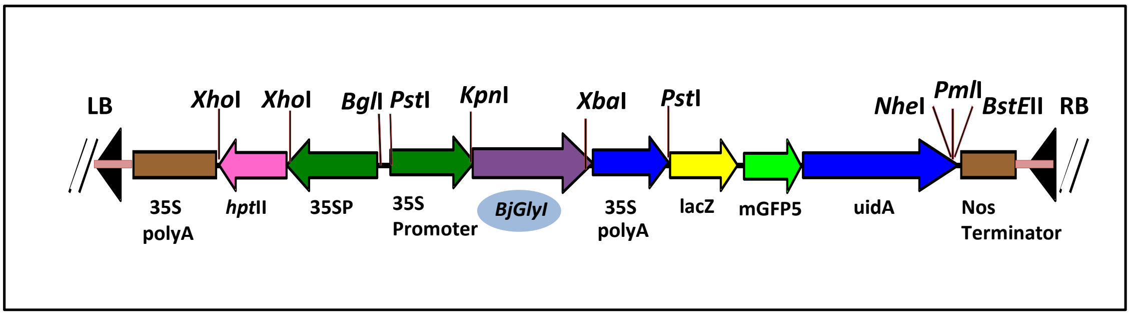

Supplement: Additional File 3 — Gene construct used for rice transformation. Schematic representation of the gene construct which shows BjGlyI cloned in pCAMBIA1304 plant transformation vector and used for Agrobacterium mediated rice transformation. [file 1746-4811-7-49-S3.PDF]
