# Supplementary material for: An improved protocol for efficient transformation and regeneration of diverse indica rice cultivars
Source: Plant Methods. 2011 Dec 30;7:49. doi: 10.1186/1746-4811-7-49 (PMC3284416; doi:10.1186/1746-4811-7-49)
Supplement: Additional File 4 — Optimization of the kind of gelling agent during regeneration. Table showing regeneration frequency using different gelling agents viz. agar, phytagel, phytagel and agar together, and agarose during regeneration in MSRMa and MSRMb. [file 1746-4811-7-49-S4.PDF]

Additional file 4: **Optimization of the kind of gelling agent during regeneration**. Table showing regeneration frequency using different gelling agents viz. agar, phytagel, phytagel and agar, and agarose during regeneration in MSRMa and MSRMb. For each cultivar, the differences between regeneration frequencies obtained with the use of different gelling agents were tested for statistical significance using one-way ANOVA, which confirmed that agarose is the best candidate for gelling agent ($P < 0.05$). Data shown are mean of three independent experiments.

Regeneration medium	Cultivar	* Mean Shoot Regeneration frequency (%)			
		Phytagel (0.3%)	Agar (0.8%)	Phytagel (0.3%) + Agar (0.8%)	Agarose (0.8%)
MSRMa	IR64	30	26	36	77
	PB1	27	24	32	68
	CSR10	29	24	31	72
	Swarna	29	26	32	70
MSRMb	IR64	28	23	30	64
	PB1	25	20	23	60
	CSR10	26	20	24	54
	Swarna	26	22	24	55

* The calli were shifted to regeneration medium, either MSRMa or MSRMb containing the gelling agents as indicated above and were allowed to regenerate under dark conditions during the first phase of regeneration (for the first 7 days) and then under light conditions during the second phase (for the next 4 days).
