# Supplementary material for: An improved protocol for efficient transformation and regeneration of diverse indica rice cultivars
Source: Plant Methods. 2011 Dec 30;7:49. doi: 10.1186/1746-4811-7-49 (PMC3284416; doi:10.1186/1746-4811-7-49)
Supplement: Additional File 5 — Optimization of proportion of hormones and agarose concentration during different phases of regeneration. Table showing regeneration frequency using various agarose concentrations in MSRMa (A) and MSRMb (B). [file 1746-4811-7-49-S5.PDF]

Additional file 5: **Optimization of proportion of hormones and agarose concentration during different phases of regeneration.**

A. Table showing regeneration frequency using various agarose concentrations in MSRMa.

Cultivar	Replicates	No. of seeds inoculated	No. of embryogenic calli generated	Total no. of calli used for regeneration [a]	Shoot Regeneration frequency (%)[b]			
					0.8% agarose	1% agarose	1% and 0.4% agarose [c]	1% and 0.8% agarose [d]
IR64	R1	200	184	552	78	70	51	88
	R2	150	142	426	73	65	47	90
	R3	180	160	480	80	63	53	83
PB1	R1	150	140	420	63	53	43	81
	R2	180	173	519	66	56	49	87
	R3	100	93	279	69	58	44	84
CSR10	R1	200	194	582	68	55	49	87
	R2	150	144	432	75	58	51	92
	R3	150	144	432	71	62	52	91
Swarna	R1	100	93	279	66	64	51	89
	R2	150	140	420	70	66	48	92
	R3	180	137	419	72	61	53	87

[a]The embryogenic calli generated after seed-inoculation were sub-cultured and used for regeneration. The sub-cultured calli were equally distributed in four sets and each set was allowed to regenerate in MSRMa with different agarose concentration as mentioned above. For details see Methods and Results.

[b]Shoot regeneration frequency (%) = No. of microcalli regenerating shoots/no. of microcalli incubated X 100%

[c]1% agarose was used during the first phase of regeneration and 0.4 % in the second phase (see Methods section)

[d]1% agarose was used during the first phase of regeneration and 0.8 % in the second phase (see Methods section)

B. Table showing regeneration frequency using various agarose concentrations in MSRMb.

Cultivar	Replicates	No. of seeds inoculated	No. of embryogenic calli generated	Total no. of calli used for regeneration [a]	Shoot regeneration frequency(%)[b]			
					0.8% agarose	1% agarose	1% and 0.4% agarose [c]	1% and 0.8% agarose [d]
IR64	R1	180	175	525	66	50	47	77
	R2	160	151	453	68	56	44	84
	R3	150	142	426	59	54	45	80
PB1	R1	150	143	429	60	45	40	72
	R2	180	170	510	57	48	43	71
	R3	150	141	423	64	50	41	76
CSR10	R1	170	155	465	52	41	39	67
	R2	150	146	438	57	44	37	74
	R3	170	162	486	54	46	34	70
Swarna	R1	160	154	462	52	49	35	74
	R2	150	142	426	57	56	38	78
	R3	160	151	453	55	52	41	71

[a]The embryogenic calli generated after seed-inoculation were sub-cultured and used for regeneration. The sub-cultured calli were equally distributed in four sets and each set was allowed to regenerate in MSRMb with different agarose concentration as mentioned above. For details see Methods and Results.

[b]Shoot regeneration frequency (%) = No. of microcalli regenerating shoots/no. of microcalli incubated X 100%

[c]1% agarose was used during the first phase of regeneration and 0.4 % in the second phase (see Methods)

[d] 1% agarose was used during the first phase of regeneration and 0.8 % in the second phase (see Methods)
